# A new remarkable subterranean beetle of the Rhodopes: *Paralovricia* gen. n. beroni sp. n. belonging to Lovriciina new subtribe (Coleoptera, Carabidae, Trechinae, Bembidiini)

**DOI:** 10.3897/zookeys.117.1612

**Published:** 2011-07-09

**Authors:** Pier Mauro Giachino, Borislav Guéorguiev, Dante Vailati

**Affiliations:** 1 Settore Fitosanitario Regionale, Environment Park, Palazzina A2, Via Livorno, 60 - 10144 Torino, Italy; 2 National Museum of Natural History, 1 Blvd. Tzar Osvoboditel, 1000 Sofia, Bulgaria; 3 Via Interna 8, 25127 Brescia, Italy

**Keywords:** Coleoptera, Carabidae, new species, new genus, new subtribe, Lovricia, Neolovricia, Rhodopes, Bulgaria

## Abstract

*Paralovricia* **gen. n.** *beroni* **sp. n.**, belonging to the new subtribe Lovriciina, is described from two caves in the Western Rhodopes (Bulgaria): Kraypatnata peshtera, near Smilyan Village (Smolyan Municipality) and Snezhanka, near Peshtera Town (Peshtera Municipality). Three currently known genera, *Lovricia* Pretner, 1979, *Neolovricia* Lakota, Jalžić & Moravec, 2009 and *Paralovricia* **gen. n.** represent a monophyletic unit supported by important synapomorphies that allows to propose the establishment of the new subtribe Lovriciina, characterized by the following characters: last maxillary palpomeres very long and narrow, basal angles of pronotum without seta, elytra without discal setae, elytral umbilicate series of nine pores in which the main pores are the 2nd, 6th and 9th, apical recurrent striole absent and mesotibial apex expanded outward. Its systematic position within the subfamily Trechinae (sensu Lorenz 2005) is discussed and Lovriciina is placed near Anillina. Key to the genera of the subtribe is proposed. Distribution data and zoogeographical hypotheses are discussed.

## Introduction

The genera *Lovricia* Pretner, 1979 and *Neolovricia* Lakota, Jalžić & Moravec, 2009 are sporadically and restrictedly distributed in Dinaric Alps. To date they contain three very rare species known only after few specimens. *Lovricia jalzici* Pretner, 1979 is presently known only for a single female specimen from cave Gospodska pećina in the vicinity of the village Cetina (Dinara Mt.); *Lovricia aenigmatica* Lakota, Mlejnek & Jalžić, 2002, for one male and one female from an unnamed pit near the peak Sveti Jure on the Biokovo Mt., which recently received the name of Lovrićija Jama I (Bedek et al. 2006) and for another female from Lovrićija Jama II (Sveti Jure, Biokovo) (Lakota et al. 2009); *Neolovricia ozimeci* Lakota, Jalžić & Moravec, 2009, for one female from cave Špilja u Radinovcima, near Dedići, Rastovac on the massif of Biokovo.

The systematic position of *Lovricia*, and its related genus *Neolovricia*, remains controversial. Pretner (1979) placed *Lovricia* as an independent genus of uncertain position followed by Casale and Laneyrie (1982) who lists it at the end of the subfamily Trechinae among “the genera with uncertain taxonomic position” but probably related with Anillini. Lakota et al. (2002), within the description of *Lovricia aenigmatica*, maintain the same approach, while in the later work concerning the description of the genus *Neolovricia* (Lakota et al. 2009), emphasize some probable relationships of these genera with Anillini (sensu Jeannel 1937). The allocation of a proper systematic position was hampered by the unknown male genital morphology. The only known male (of *Lovricia aenigmatica*) was an immature specimen and its genitalia were damaged during the extraction (Lakota et al. 2002).

The discovery, by Petar Beron, Boyan Petrov and Pavel Stoev, in two different caves in the Rhodopes, of two specimens (male and female) of a new genus and new species clearly belonging to the same phyletic lineage (originally mentioned as “undefined blind carabid beetle” by Giachino and Guéorguiev 2006: 61) and as “Trechini gen. & sp. indet.” by Guéorguiev and Lobo (2006: 305), allows us to clarify the systematic position of this monophyletic complex.

## Material and methods

The following acronyms have been used for depositories of material:

NMNHSNational Museum of Natural History, Sofia, Bulgaria
            

CGiCollection Giachino, Torino, Italia.
            

The following acronyms have been used for the type material:

HTHolotype
            

PTParatype
            

The specimens, whose drawings of the habitus and genitalia were made, were previously included in Canada balsam. Genitalia were pinned beneath the specimens. The drawings were made by means of a camera lucida connected to a Leitz Dialux. Measurements in millimetres (mm).

For measurement uniformity it was decided to use the same methodology proposed by Sokolov et al. (2004), so measurements for various body parts are coded as follows:

ABLapparent body length, from clypeus to apex of elytra;
            

WHhead width, at the level of the first orbital setae;
            

WPmmaximum width across pronotum;
            

WPawidth across anterior angles of pronotum;
            

WPpwidth across posterior angles of pronotum;
            

LPlength of pronotum from base to apex along midline;
            

WEmaximum width of elytra;
            

LElength of the elytra, from apex of scutellum to apex of left elytron.
            

Only ABL measurements are reported, the other measurements are given as 7 ratios.

General width: WH/WPm and WPm/We.

Body parts: WPa/WPp, WPm/WPp, WPm/LP and WE/LE.

### Paralovricia gen. n.

urn:lsid:zoobank.org:act:AA3230A9-D4BC-45C8-BC8F-0CEF2688F108

http://species-id.net/wiki/Paralovricia

#### Type species:

*Paralovricia beroni* sp. n.

#### Diagnosis.

A genus closely related to *Lovricia* and *Neolovricia* by the shape of the penultimate maxillary palpomeres, narrow and elongate, by the hind angle of the pronotum lacking seta, and by mesotibiae distally expanded outwards. *Paralovricia* gen. n. differs from both *Lovricia* and *Neolovricia* by the less “aphaenopsian” body shape, with a more cordiform pronotum and elytra with more evident and finely denticulate shoulders. From *Lovricia*, the new genus differs by the shape of the penultimate maxillary palpomere not subtriangular and by mesotibiae furnished of spurs besides the inner angles. From *Neolovricia*, the new genus differs by the labium with a developed median tooth and by the different shape of the female gonocoxite.

#### Description.

Body small (ABL = mm 1.80 - 2.19), elongate, rather flattened, anophthalmous, pubescent, reddish-ferrugineous, with palpi and last tarsomeres paler.

Head relatively large, clypeus separated from the frons by a transverse impression, with two pairs of long thin setae. Frontal furrows ending with round foveae impressed posteriorly, neck distinct; two supraorbital setae not reduced in length. Labrum transverse, with a slight emargination in the middle, bearing six setae. Mandibles slender with a simple apex. Maxillae strongly prominent, penultimate segments of maxillary palpi longer than broad, slightly clavate, terminal palpomeres protracted, needle-shaped and pointed. Mentum without a suture between labium and pre-basilar; labium with a large median tooth. Antennae moniliform. Surface fine, microreticulate, pubescence relatively dense, recumbent, as long as that of pronotum and elytra. Cephalic capsule covered by yellowish, recumbent, relatively long and sparse hairs.

Pronotum subcordiform, hardly explanate sides usually narrowed at posterior angles, with the maximum width at the anterior third. Anterior edge arcuate, with angles entirely rounded. Lateral margin hardly sinuated before rectangular posterior angles. Disc slightly convex, with longitudinal median furrow impressed. Basal transversal furrow deep and conspicuous. Surface with distinct puncturation and long, somewhat sparse, fine erected pubescence. Anterior sixth of their length with pair of marginal setae; basal setae lacking. Scutellum subtriangular.

Elytra longer than their combined width, rounded apically near the suture, wholly covering the abdomen, dorsoventrally slightly convex without a distinct marginal groove. Humeral angles rounded but evident; lateral margins with edges finely denticulate. Sculpture of elytra distinctly microreticulate consisting of wrinkled lines; striae missing; recurrent striola lacking. Elytral disc without discal setiferous punctures, with pubescence relatively dense, recumbent and long, not arranged in rows. Umbilicate series consisting in 9 setiferous pores, with 2nd, 6th and 9th ones bearing a long seta; a geminate pair is made by 5th and 6th pores, with the 5th placed after the 6th one.

Abdominal sterna smooth, with fine and sparse pubescence.

Legs relatively short. Femora robust. Protarsomeres not dilated in the male. Mesotibiae distally expanded outwards and fringed with dense bristles, inner angles with additional spurs. Metafemora smooth. Tarsal claws simple.

Aedeagus with the median lobe stout and poorly arcuate, with a simple narrowly and irregularly sub-squared apex. Basal bulb with the orifice greatly expanded dorsally and delimiting two subequal lateral lobes as in Anillini (Jeannel 1941). Left and right parameres similar to each other, long, widened at the base; narrow, elongated and strongly curved upwards at the apex. One large coaxial seta at the apex and a second one, small, ventral, in a preapical position. Inner sac with a median copulatory sclerite, clew-shaped with two dorsolateral branches.

Female genitalia with the gonocoxite separated from the subgonocoxite (Fig. 11). The latter narrow and elongated, drop-like shaped, totally free from setae or thorns. Gonocoxite stocky, angular and slightly curved, not pointed at the apex; bearing 3 stocky ensiform setae on the external-proximal edge and a dorsal one at the internal edge. The apical part of the gonocoxite shows, at the inner edge, a sensorial dimple bearing two fine and short nematiform setae. Spermatheca ((Fig. 10) short and stocky, membranous, not sclerotized; spermathecal gland not found.

#### Etymology.

*Paralovricia* (feminine in gender), combination of the Greek prefix “παρα” (= near) and the genus name *Lovricia*.

### Paralovricia beroni sp. n.

urn:lsid:zoobank.org:act:FFDDD4DB-5B2E-4036-9DD2-C30C3A14A347

http://species-id.net/wiki/Paralovricia_beroni

Figs 1 [Fig F2] -11 

#### Type locality:

Bulgaria, Western Rhodopes, Smolyan Municipality, near the village of Smilyan, Kraypatnata peshtera cave, 41.5123° N; 24.7600° E, 780 m.

#### Type series:

HT ♂, Bulgaria, Western Rhodopes, Smolyan Municipality, near the village of Smilyan, Kraypatnata peshtera cave, 41.5123° N; 24.7600° E, 780 m, 11.VII.1997, Boyan Petrov leg. (NMNHS). PT: 1 ♀, Bulgaria, Western Rhodopes, Peshtera Municipality, near the town of Peshtera, Snezhanka cave, 42.0092° N; 24.2720° E, 860 m, 17.VI.2005, Petar Beron & Pavel Stoev leg. (CGi).

Note: Male HT was completely dismembered and lacking of abdominal sternites and left metathoracic leg. The drawing of the habitus of this specimen is therefore entirely reconstructed on the basis of individual anatomical parts that are included now in Canada Balsam.

#### Description:

Body small (ABL = mm 1.80 ♂ 2.19 ♀), elongate, rather flattened, anophthalmous. Pubescence very sparse, short, yellow, recumbent.

Head relatively large but narrower than pronotum (WH/WPm = 0.97 ♂, 0.95 ♀), clypeus truncate with the frontoclypeal sulcus distinct. Frontal furrows with posterior round foveae, occiput coarsely and densely punctate. Mandibles slender with a simple apex. Maxillae strongly prominent, penultimate segments of maxillary palpi longer than broad, bearing 4 setae, terminal palpomeres protracted, needle-shaped, with an apical tuft of sensillae (Figs 3-4). Labium (Figs 5-6) with a large median tooth, showing two small basal setae; mentum with a large, rounded, depressed fovea, latero-posteriorly surrounded by a ring of 10-12 setae. Antennae moniliform from the fourth antennomere onwards, long, markedly exceeding the humeral portion of the elytra when stretched backwards. Cephalic chaetotaxy as in the description of the genus.

Pronotum slightly convex, subcordiform (WPa/WPp = 1.42 ♂, 1.54 ♀), with the maximum width at the anterior third (WPm/LP = 1.09 ♂, 1.10 ♀). Anterior angles obtuse and broad. Lateral margin hardly sinuated before the posterior angles, which are rectangular and slightly projecting laterally. Punctures of the disc nearly equal to those of the occiput. Anterior sixth of their length with a pair of marginal setae; basal setae lacking. Scutellum subtriangular, pointed apically, with distinct transverse cells.

**Figures 1-2.**
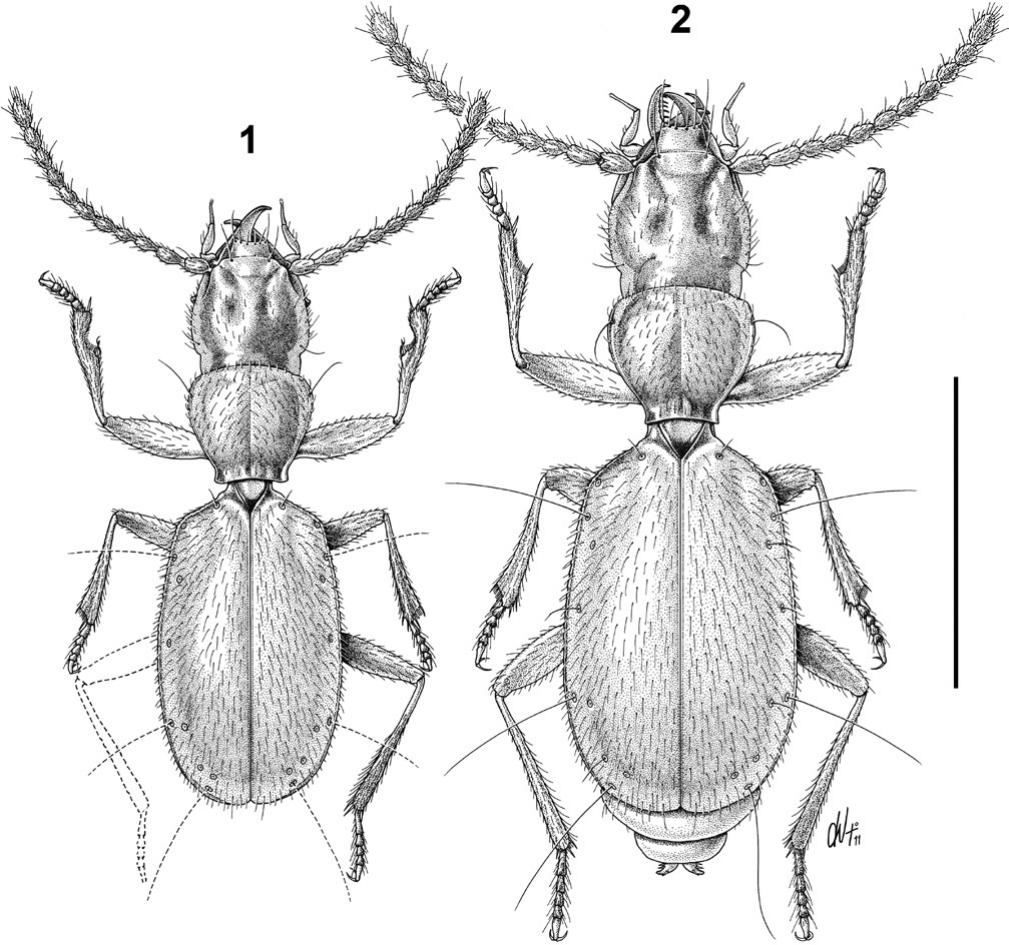
Habitusof *Paralovricia* gen. n. *beroni* sp. n. **1** HT ♂ **2** PT ♀. Scale bar: 1 mm.

Elytra longer than their combined width (WE/LE = 0.62 ♂, 0.66 ♀), widest closely behind one half of their length. Humeral angles rounded but evident; lateral margins without a distinct marginal groove but with edges finely denticulate. Sculpture of elytra distinctly microreticulate consisting of wrinkled lines; striae missing; recurrent striola lacking, disc without discal setiferous punctures. Scutellar pore umbilicate and shifted from its normal position, placed near the front edge of the elytra. Umbilicate series as in Figs 1-2, consisting in 9 setiferous pores; the main umbilicate pores bearing a long seta (sensu Giachino and Vailati in press) are the 2nd, 6th and 9th ones. 5th and 6th pores make a geminate pair, 5th, 7th and 8th decidedly shifted on the disc; 5th pore shifted after the 6th one.

Protarsomeres not dilated in the male. Mesotibiae (Figs 7-8) distally expanded on outwards and fringed with dense bristles, inner angles with additional spurs. All the last tarsomeres of pro- meso- and metatibiae hyaline and with a peculiar shape: widened at the base and narrowed at the apex.

Aedeagus (Fig. 9) with median lobe stout and poorly arcuate; apex, in lateral view, stout, and irregularly sub-squared, slightly bent downwards. Basal bulb of the median lobe small, with the basal orifice greatly expanded dorsally, reaching about one third of the length of the median lobe, delimiting two subequal lateral lobes. Shape of left and right parameres similar to each other, long, strongly widened at the base, sharply restricted, elongated and strongly curved upwards in the apical third. One large and stout coaxial seta at the apex and a second one, frail, small, ventral, in a preapical position. Inner sac with a median copulatory sclerite, clew-shaped with two dorsolateral branches.

Female genitalia as in the description of the genus.

#### Etymology:

This interesting new species is dedicated to one of its collectors, Dr. Petar Beron, a passionate biospeleologist, former Director of the National Natural History Museum of Sofia and former Vice-President of the Bulgarian Parliament, as a sign of friendship and esteem for the impetus given to the knowledge of the Bulgarian subterranean fauna.

#### Distribution and ecology.

*Paralovricia beroni* gen. n. sp. n. was discovered in the cave Kraypatnata peshtera (in English: “cave near the way”). The cave (Fig. 12) is situated on the left riverbank of the river Arda, at an altitude of 780 m a.s.l. and approximately 1 km east of the village of Smilyan. It is a diaclase cave with a total length of 38 m, -10 m in depth, and a precipice at the end. The cave entrance is situated about 2-3 meters above the level of the road Smilyan-Rudozem. Air temperature measured in the last chamber is 12°C. The cave has an ascending principal gallery, dripping water in some places and the floor covered with wet clay, rotten logs, and some bat guano. The beetle fauna there consists of *Laemostenus plasoni plasoni* (Reitter, 1885) and the Leptodirine *Gueorguievella petrovi* Giachino & Guéorguiev, 2006 (Giachino and Guéorguiev 2006). In this cave the male specimen of *Paralovricia beroni* gen. n. sp. n. was found digging in rotten wood.

The second known locality (Fig. 12), cave Snezhanka (in English: “Snow-White”) is a national tourist site. This cave is provided with utilities and has limited access to the interior. The cave is situated 5 km southwest of the town of Peshtera, on the left slope over the Novomachlenska reka River, a tributary of the Stara reka River (Petrov and Stoev 2007). It has a total length of 368 m (in the main axis 145 m) and a depth of -18 m. The main chamber measures 48 × 36 m. The female of the new species has been collected in a small right side-gallery immediately after the entrance; this part of the cave is unlit and normally not visited by tourists. The entrance is situated in the midst of a beech forest (*Fagetum sylvatica*). The beetle fauna inside includes as well *Bryaxis* sp. (R. Bekchiev det.).

It is worth mentioning that the distance between these two caves is 64-65 km by airline (Fig. 12) and that the same species lives in such a relatively wide distance. This is not only a remote question, but between these points are situated the valley of Vacha River and first and third highest elevations of the Rhodopes. Chernatitsa Mt. (with maximal point Golyam Persenk, 2091 m a.s.l.) in the north and the Perelik Mt. (with maximal point Golyam Perelik, 2191 m a.s.l.) in the south form united mountain ridge with lowest points between them the col Pamporovo (1620 m a.s.l.) and the col Prevala (1665 m a.s.l.). This seems to confirm that the apparent rarity of one species cannot be presumed as synonymous of short range distribution. It may be attributed instead to our lack of bionomic knowledge. Indeed, both caves were visited several times by biospeleologists at any time, but no more specimens from this new genus have been found. For instance, after finding of this new species, the Kraypatnata peshtera Cave was visited six times, and the Snezhanka Cave more than ten times after that. According to Lakota et al. (2002), the species of *Lovricia* are very rare because of their hidden bionomy. For the time being, we have very scanty information on the life history of these remarkable beetles. It seems very probable that *Paralovricia beroni* sp. n. just like most known Anillini, is not typical cave-inhabitant. It lives, probably, in the deep network of microcaverns and cracks, as supposed by Giachino and Vailati (2010) for many subterranean beetles, from where penetrates accidentally into people-accessible caves.

#### Systematic discussion.

As already discussed in the introduction, the systematic position of the genera complex formed by *Lovricia* and *Neolovricia*, to which now *Paralovricia* gen. n. is added, has always been controversial. The lack of knowledge on the morphology of the aedeagus, even in a single known species, together with a too brief, too superficial, or misinterpreted description, of a number of important characters, such as the elytral chaetotaxy, helped to postpone the solution of the problem. In this way, some important phylogenetic characters could not be controlled with certainty because they were misinterpreted or omitted from the original descriptions. For example, we do not know if, even in *Lovricia* and *Neolovricia*, scutellar setiferous pores are moved toward the elytral base. While, conversely, an examination of the original drawings, although incomplete (in small specimens drawn without inclusion in Canada Balsam), allows us to say with good approximation that the umbilicate series of *Lovricia* and *Neolovricia* are similar to that of *Paralovricia*.

The three currently known genera, *Lovricia*, *Neolovricia* and *Paralovricia* represent a clear monophyletic unit supported by important synapomorphies that allow us to propose the establishment of a new subtribe.

**Figures 3-8. F2:**
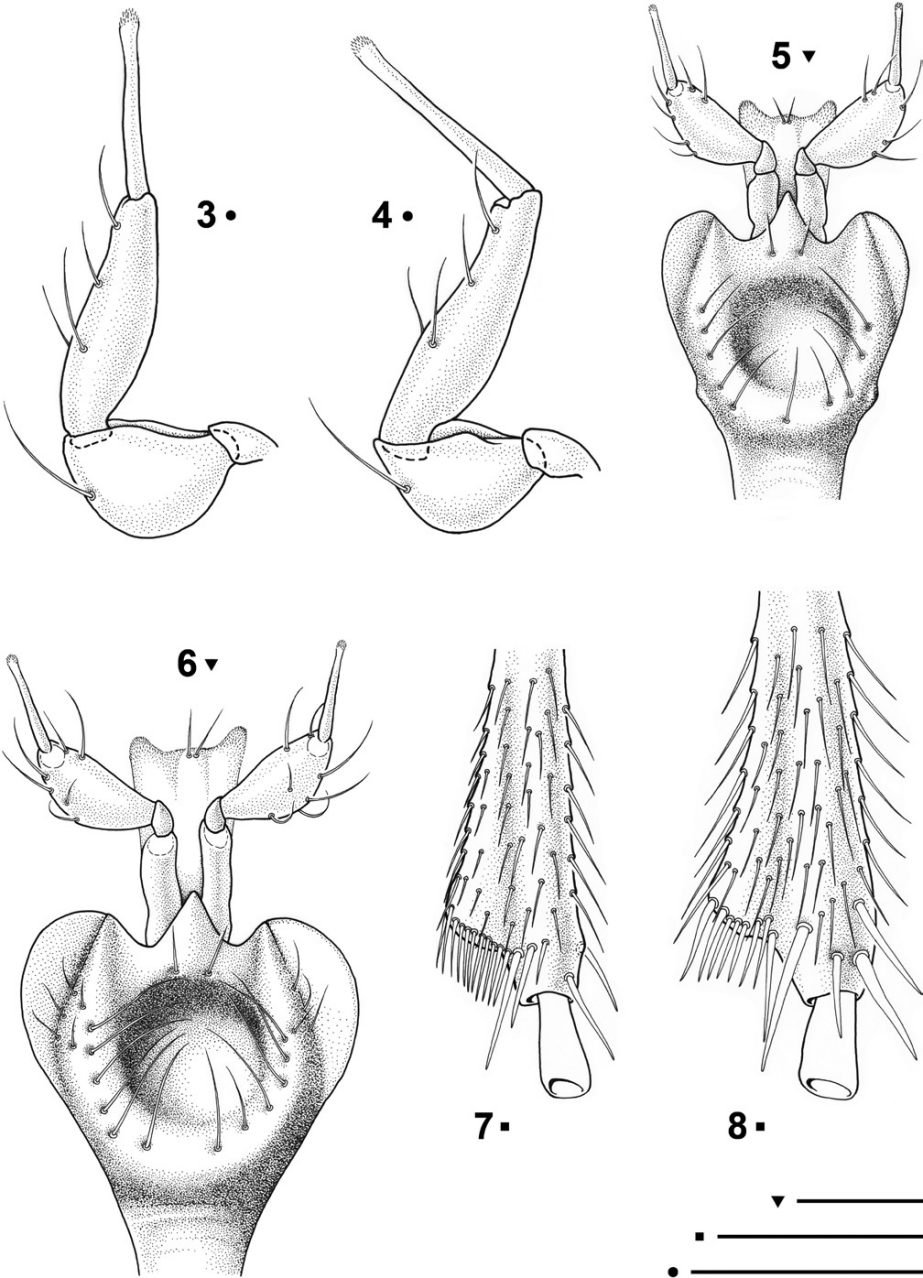
*Paralovricia* gen. n. *beroni* sp. n. **3** Maxillary palpomere, HT ♂ **4** Maxillary palpomere, PT ♀ **5** Mentum, labial palpomeres and ligula in ventral view, HT ♂ **6** Mentum, labial palpomeres and ligula in ventral view, PT ♀ **7** Apex of right mesotibia in ventral view, HT ♂ **8** Apex of right mesotibia in ventral view, PT ♀. Scale bars: 0.1 mm.

**Figures 9-11.**
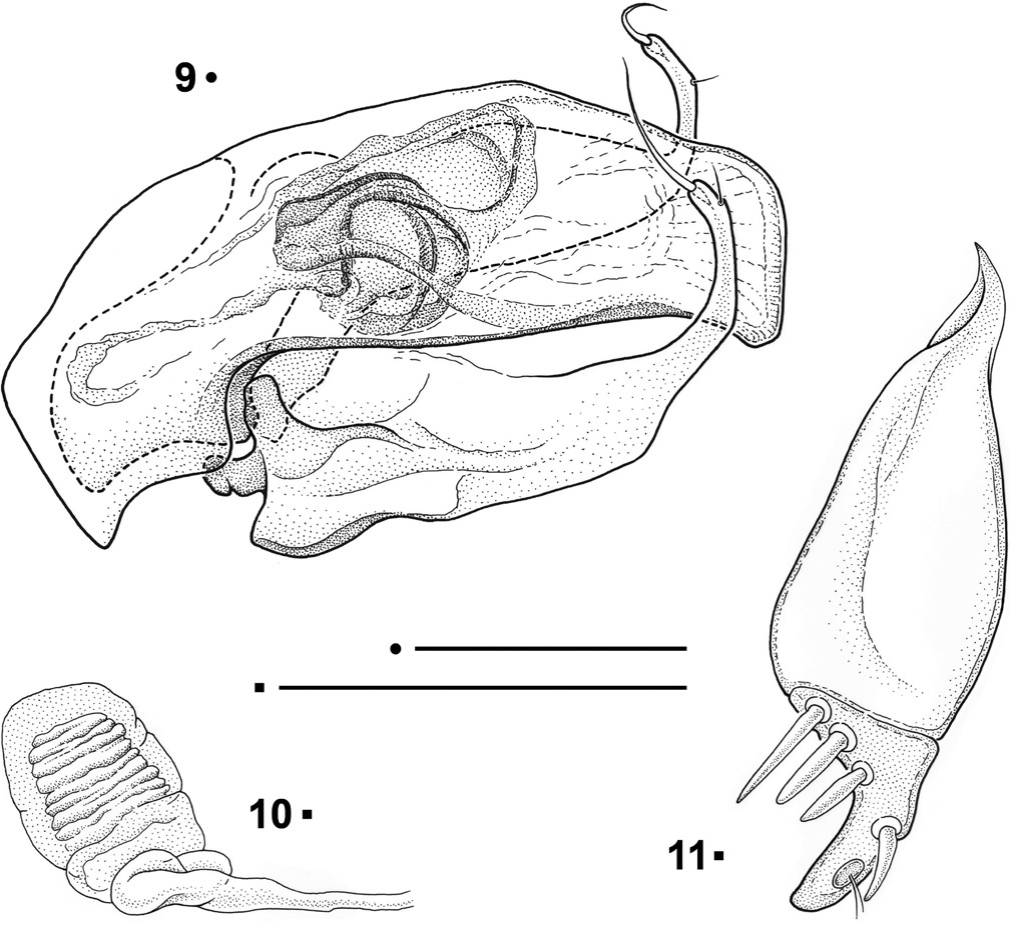
*Paralovricia* gen. n. *beroni* sp. n. **9** Aedeagus in lateral view, HT ♂ **10** Spermatheca, PT ♀ **11** Right gonocoxite in ventral view, PT ♀. Scale bars: 0.1 mm

**Figure 12.**
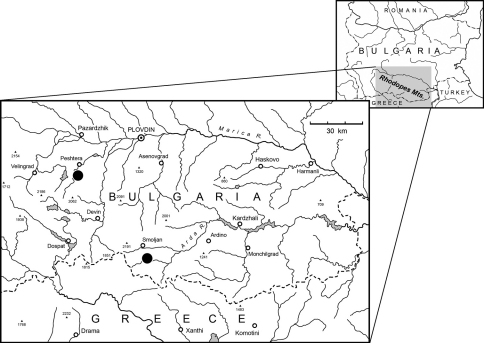
Distribution map of genus *Paralovricia* gen. n. (black circles indicate two known findings of *Paralovricia beroni* sp. n.).

### Lovriciina subtrib. n.

http://species-id.net/wiki/_Lovriciina

#### Type genus.

*Lovricia* Pretner, 1979

#### Diagnosis.

A subtribe characterized by genera that present the following synapomorphic characters: last maxillary palpomeres very long and narrow, basal angles of the pronotum without seta, elytra without discal setiferous punctures, elytral umbilicate series of nine pores in which the main pores are the 2nd, 6th and 9th ones, apical recurrent striole absent and mesotibial apex expanded outward.

#### Systematic position.

Now the examination of several characters allows us to define better the position of Lovriciina within the subfamily Trechinae (sensu Lorenz 2005). The initial doubt about the attribution of *Lovricia* to Trechini rather than Bembidiini was given by the strange morphology of the last maxillary palpomeres, which do have neither the typical conical shape of Trechini nor the small and very reduced one of Bembidiini. The examination of the elytral umbilicate series consisting in 9 setiferous pores as in Bembidiini (Jeannel 1941) can now exclude, with certainty, the belonging of Lovriciina to Trechini, that is characterized instead by a series of 8 umbilicate pores (Jeannel 1941). The absence, in Lovriciina, of an apical recurrent striole on the elytra, which is present in Bembidiina and Tachyina but lacking in Anillina (Jeannel 1941), as well as the basal part of the median lobe of the aedeagus, divided into two sub-equal basal lobes, characteristic of Anillina (Jeannel 1941), allows us to assign Lovriciina near Anillina (sensu Lorenz 2005).

#### Key of the genera of Lovriciina

**Table:** 

1	Pronotum cordate, wider than long (ratio pronotum width / pronotum length more than 1.05)	*Paralovricia* gen.n.
–	Pronotum sub-elongate, longer than wide (ratio pronotum width / pronotum length less than 0.95).	2
2	Smaller species (length of body less than 2.2 mm). Shoulders of elytra distinct, more or less angulate.	*Neolovricia* Lakota, Jalžic & Moravec, 2009
-	Larger species (length of body more than 2.3 mm). Shoulders of elytra indistinct, oblique, without distinct angles.	*Lovricia* Pretner, 1979

#### Zoogeography.

**Figure 13.**
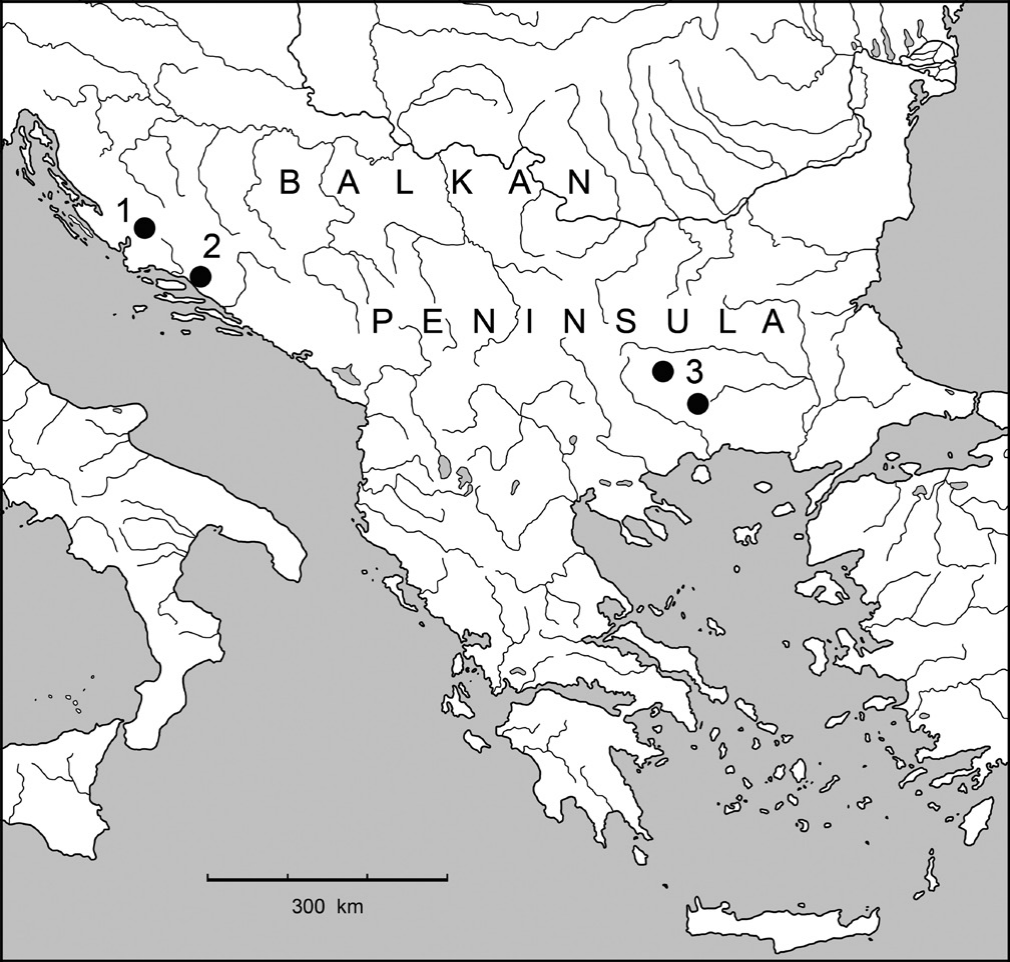
Distribution map of the species from subtribe Lovriciina (1 – *Lovricia jalzici* Pretner; 2 – *Lovricia aenigmatica* Lakota, Mlejnek & Jalžić and *Neolovricia ozimeci* Lakota, Jalžić & Moravec; 3 – *Paralovricia beroni* sp. n.).

Analysis, from a historical zoogeographical point of view, of the distribution of the phyletic lineage of Lovriciina (Fig. 13) provides several interesting insights. First we must consider that the currently known distribution, although widely disjoint, ranging from the Dinarides to the Rhodopes and that, as widely discussed for Anillina (Giachino 2005, 2008, Giachino and Vailati in press), we are handling a group with a likely ancient origin. In this way we must go back at least to the Late Oligocene (29-24 Ma) before finding, in the paleogeographic reconstructions currently available (Popov et al. 2004), a continuum of land that connects each other Dinarides and Rhodopes allowing a colonization by this phyletic lineage. Conversely, a paleogeographic event that could be placed at the origins of the separation of *Paralovricia* (on Rhodopes) from a common ancestor, which then enabled a further differentiation of *Lovricia* and *Neolovricia* on Dinarides, may be identified in the Early Miocene (20.5-19 Ma) when a strip of lowlands, covered with freshwater lakes and marshes seems to have again divided the Dinarides from Rhodopes (Popov et al. 2004).

## Supplementary Material

XML Treatment for Paralovricia

XML Treatment for Paralovricia beroni

XML Treatment for Lovriciina
